# Marine protected areas do not buffer corals from bleaching under global warming

**DOI:** 10.1186/s12862-022-02011-y

**Published:** 2022-05-04

**Authors:** Jack V. Johnson, Jaimie T. A. Dick, Daniel Pincheira-Donoso

**Affiliations:** 1 Macrobiodiversity Lab, School of Biological Sciences, Queen’s University Belfast, 19 Chlorine Gardens, Belfast, BT9 5DL UK; 2 Institute for Global Food Security, School of Biological Sciences, Queen’s University Belfast, 19 Chlorine Gardens, Belfast, BT9 5DL UK

**Keywords:** Antagonistic, Anthropocene, Climate change, Coral reefs, Degradation, Local stressors, MPA, Scleractinia

## Abstract

**Background:**

The rising temperature of the oceans has been identified as the primary driver of mass coral reef declines via coral bleaching (expulsion of photosynthetic endosymbionts). Marine protected areas (MPAs) have been implemented throughout the oceans with the aim of mitigating the impact of local stressors, enhancing fish biomass, and sustaining biodiversity overall. In coral reef regions specifically, protection from local stressors and the enhanced ecosystem function contributed by MPAs are expected to increase coral resistance to global-scale stressors such as marine heatwaves. However, MPAs still suffer from limitations in design, or fail to be adequately enforced, potentially reducing their intended efficacy. Here, we address the hypothesis that the local-scale benefits resulting from MPAs moderate coral bleaching under global warming related stress.

**Results:**

Bayesian analyses reveal that bleaching is expected to occur in both larger and older MPAs when corals are under thermal stress from marine heatwaves (quantified as Degree Heating Weeks, DHW), but this is partially moderated in comparison to the effects of DHW alone. Further analyses failed to identify differences in bleaching prevalence in MPAs relative to non-MPAs for coral reefs experiencing different levels of thermal stress. Finally, no difference in temperatures where bleaching occurs between MPA and non-MPA sites was found.

**Conclusions:**

Our findings suggest that bleaching is likely to occur under global warming regardless of protected status. Thus, while protected areas have key roles for maintaining ecosystem function and local livelihoods, combatting the source of global warming remains the best way to prevent the decline of coral reefs via coral bleaching.

**Supplementary information:**

The online version contains supplementary material available at 10.1186/s12862-022-02011-y.

## Introduction

Rising ocean temperatures and increased frequency and duration of marine heatwaves [1] are causing the decline of coral reefs at alarming rates via coral bleaching [2, 3]—the process whereby photosynthetic endosymbionts are expelled, revealing the coral skeleton [4–8]. Sustained ocean heat stress can lead to mass bleaching events which may result in mortality of entire coral colonies [3, 9, 10]. If lethal bleaching occurs, the loss of coral cover results in habitat homogenisation and consequently reduced biodiversity [10, 11]. Ultimately, such reductions in coral reef biodiversity inhibits the ecosystem function of coral reefs, critical for supporting > 25% of marine species [12] and for providing ecosystem services to over 100 million people circumtropically [13].

While global warming is unequivocally the predominant driver of mass coral bleaching, a myriad of local scale factors can also induce bleaching of corals. Factors such as turbidity [14, 15], eutrophication [16, 17], hypoxia [16, 18], and sedimentation [14] have been documented to independently induce coral bleaching. However, pioneering studies identified reduced mortality from coral bleaching under higher levels of sedimentation when exposed to heat stress, likely as a result of reduced solar irradiance [14]. Yet, bleaching is known to occur under high temperature regimes regardless of irradiance [19]. Despite convoluted evidence of these interactions influencing bleaching, a widespread expectation that these local stressors interact either additively or synergistically with global warming to exacerbate coral bleaching exists, with field evidence from Mesoamerican reefs [20, 21], and French Polynesia [17].

To mitigate the additive effects of local scale stressors on marine biodiversity overall, Marine Protected Areas (MPAs) have been implemented across different regions of the world, which when effectively designed, are established to enhance regional biodiversity and general ecosystem health [22]. Large and long-established MPAs are often especially effective for enhancing multiple metrics used for monitoring ecosystem health [23, 24], such as coral cover [25], fish biomass [26] and biodiversity [22]. For corals specifically, the role MPAs perform for reducing local stressors intend to enhance coral health through a variety of physiological mechanisms [27], thereby promoting resistance of reef building corals to disturbance. Furthermore, MPAs have the potential to promote resilience to disturbance events, such as marine heatwaves, disease outbreaks, and hurricanes, via ecological processes [28]. This enhanced resilience intrinsically promotes resistance to future bleaching by facilitating full recovery from bleaching before the next disturbance event [11]. Given that marine heatwaves and bleaching events are increasing in frequency and intensity through time [1, 2, 11, 29], the benefits of MPAs for promoting resilience in reef building corals are subsequently crucial for also enhancing the resistance of corals to future bleaching—i.e. managed resilience [22].

However, the effects of MPAs for mitigating coral reef decline remain contested. For example, decline in coral cover attributed to thermal stress is not mitigated by MPAs [30], suggesting that the preservation of coral reefs does not depend significantly on MPAs, but on actions that mitigate the degree of climate warming [22, 31]. Furthermore, multiple stressors on coral reefs tend to be antagonistic rather than synergistic, especially interactions between local stressors, and global warming [22, 32–35]. This is likely owing to co-sensitivity and co-tolerance of coral species exposed to stressors, along with the frightening prospect that climate warming eclipses the potential advantages that could be expected to result from the mitigation of local stressors [31].

**Fig. 1:**
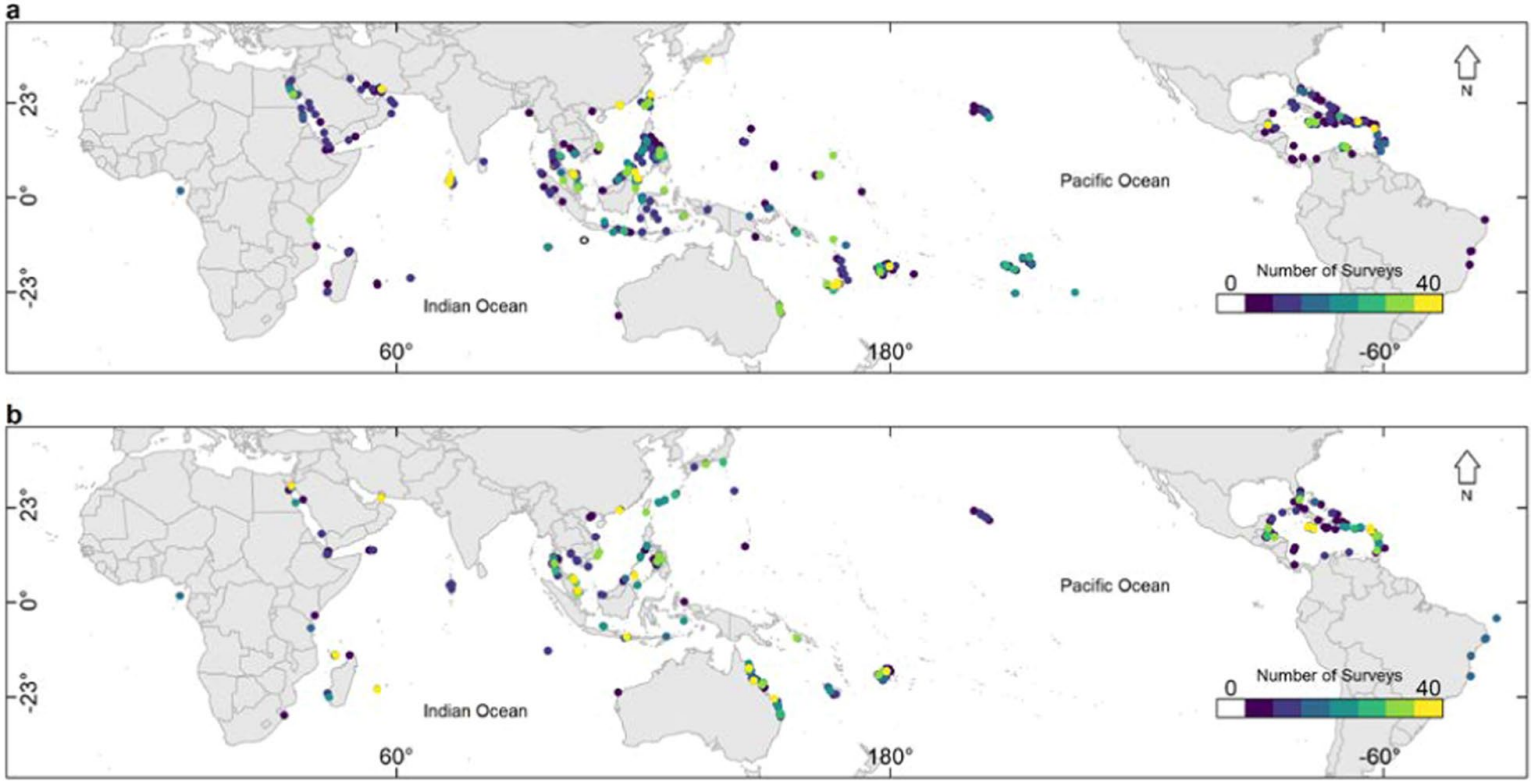
The richness and global scale distribution of Reef Check surveys used to examine the effects of MPAs on coral bleaching. **a** Represents 5393 surveys which do not fall within an MPA. **b** Are the 3391 surveys which do fall within the jurisdiction of an MPA

Given both the convoluted relationship between global and local stressors exerted on coral reefs, along with the diversity of primary objectives different MPA’s aim to achieve, it is crucial to discern whether MPA’s have any moderation effect on bleaching under global warming. However, an explicit global scale test to examine the prevalence of bleaching in relation to their protected status remains lacking. To test this hypothesis for coral bleaching specifically, we examine (1) the probability of coral bleaching under thermal stress (quantified as Degree Heating Weeks, DHW) for key MPA attributes—the size and age of MPAs, using a Bayesian Generalised Linear Mixed Model; (2) implement quantitative comparisons of bleaching prevalence on coral reefs within and outside protected regions under different levels of thermal stress; and (3) compare thermal thresholds where the onset of bleaching occurs between protected and non-protected coral reefs based on the gamma distributions of DHWs. To address this hypothesis we utilise a global scale data set containing 8,766 coral bleaching surveys (Fig. 1) over a 16 year period.

## Results

### Bleaching within MPAs

**Fig. 2:**
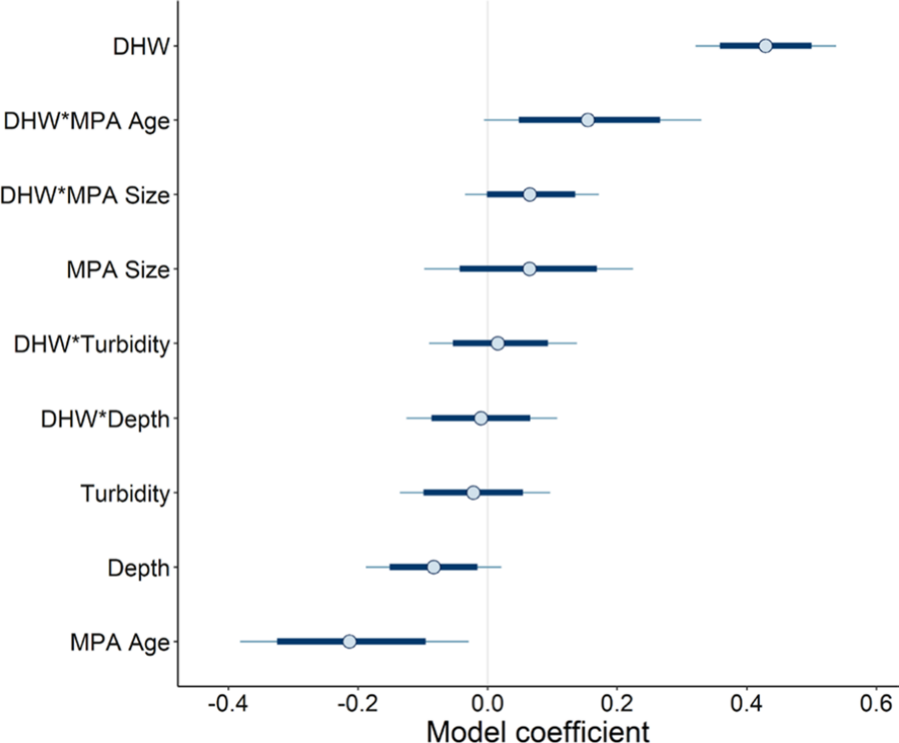
Model coefficients of predictions from the Bayesian Generalized Mixed Effect Model. DHW are degree heating weeks, MPA attributes are size and age. Blue dot represents mean coefficient, thick dark blue bars are 80% credible intervals (CI), while the whiskers (thin grey bars) are 95% CIs. Data are from 3391 coral bleaching surveys which fell within the jurisdiction of an MPA as categorized by the IUCNs World Database of Protected Areas

Our Bayesian model from 3391 reef check surveys reveals that older MPAs reduce the probability of coral bleaching, while the size of a MPA shows little evidence for predicting bleaching (Fig. 2).

However, the interaction between DHW and MPA age shows evidence for predicting an increased probability in bleaching based on the 80% credible interval (CI), which becomes weaker when interpreting the 95% CI. Meanwhile, the interaction between DHW and MPA size show little evidence for predicting bleaching as both the 80% and 95% credible intervals cross zero (Fig. 2). These interactions suggest bleaching is likely to occur when under heat stress, but do show a partial moderation in comparison to the sole predictor of DHW.

### Bleaching comparisons between MPA and non-MPA coral reefs

**Fig. 3:**
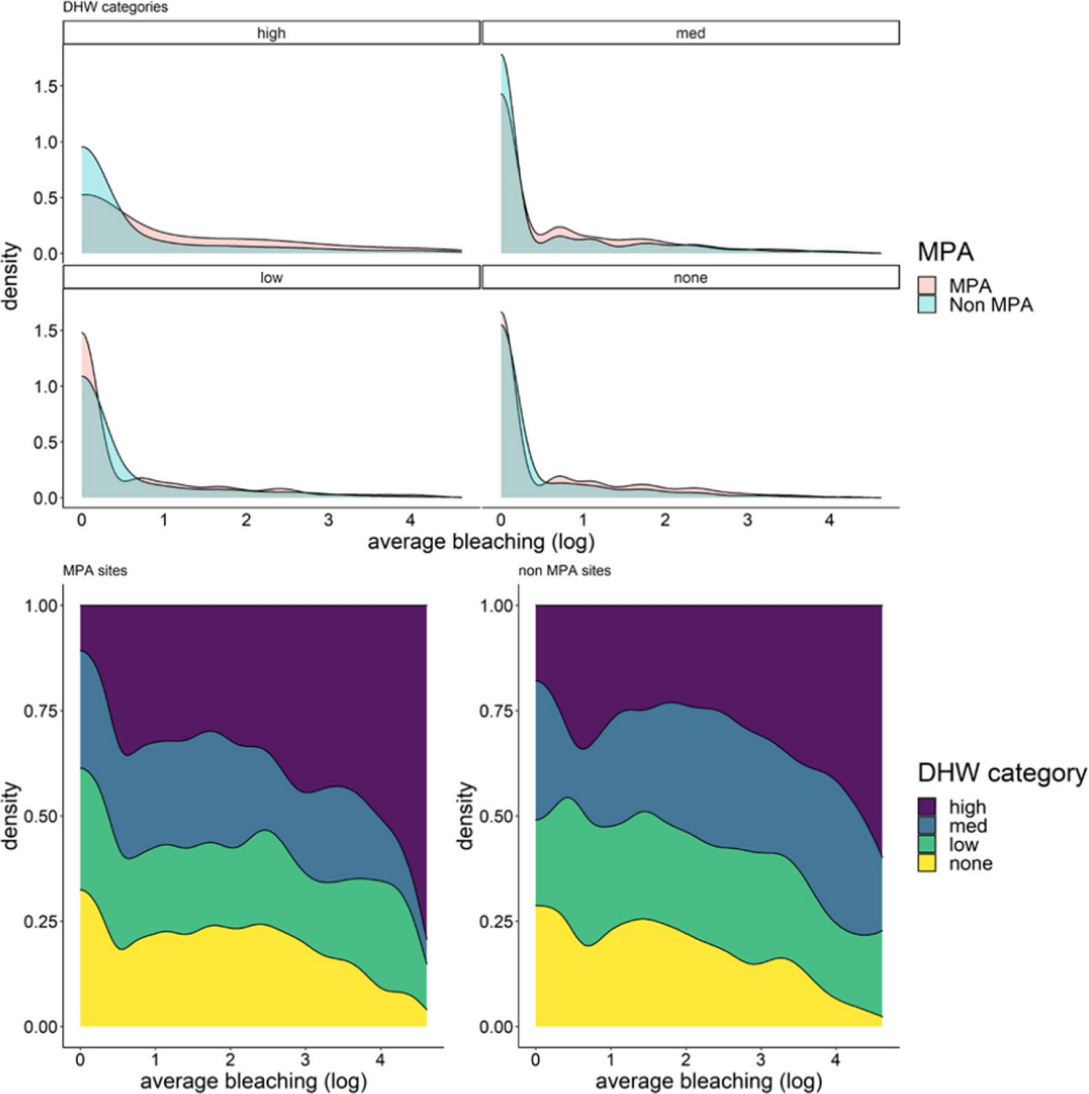
Density plots of average bleaching for each degree heating week (DHW) category; none (zero DHW), low (0–1.5 DHW), medium (1.5–4 DHW) and high (> 4 DHW). Average bleaching were log transformed for visual display purposes only. The top panel represents the distribution density of bleaching between MPA and non-MPA sites at high, medium, low, and none DHW categories. The bottom panel show the stacked proportional densities for MPA (left) and non-MPA sites (right) for each DHW category

When comparing the average bleaching prevalence for reef check surveys across different DHW categories (none 0, low < 1.5, medium 1.5–4, and high > 4) with a likelihood ratio test there were no statistically significant differences between MPA and non-MPA sites (Fig. 3; Table 1), suggesting that the sites protected status had negligible effect on bleaching prevalence.

**Table 1:** Summarised average bleaching prevalence for coral reef sites and their likelihood of being statistically different between MPA and non-MPA sites based on their thermal stress (DHW category)

	Average bleaching (%)		Likelihood ratio test
DHW category	MPA	Non-MPA	Pr (>χ2)
None	2.069	1.122	1
Low	2.573	1.859	0.985
Medium	2.369	2.347	0.999
High	6.336	3.052	1

From the likelihood ratio test, Pr(>χ2) indicates the likelihood of distributions between groups being significantly different (assuming an α level of 0.05) based on a Chi-square (χ2) test

**Fig. 4:**
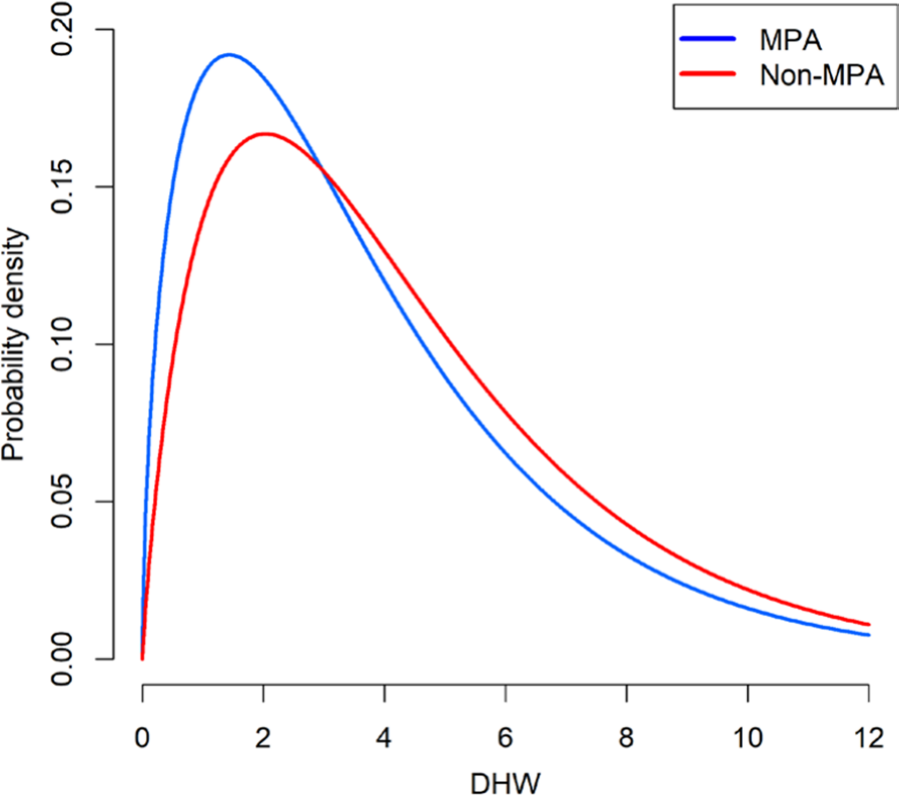
Probability densities for the degree heating week (DHW) values where the onset of coral bleaching occurred based on gamma distributions between MPA and non-MPA Reef Check survey sites. A constant of 1 was added to DHW values to meet gamma distribution assumptions where values are required to be greater than zero [36]

Finally, when comparing the DHW values where the onset of coral bleaching has occurred (i.e. the mean DHW value associated with coral bleaching) with a likelihood ratio test, there is no significant difference between MPA and non-MPA sites (Fig. 4) (Likelihood ratio test, Pr(>χ^2^) = 0.998). Thus, protected status appears to have little, if any, moderation effect on coral bleaching prevalence under thermal stress from climate change when comparing protected reefs to non-protected reefs.

## Discussion

Our analyses reveal that MPAs play a negligible role in mitigating the onset of coral bleaching when under climate change related thermal stress. However, weak evidence for moderation under the interaction of DHW with MPA size, and MPA age, in comparison to the sole predictor of DHW does exist (Fig. 2). Crucially, however, bleaching is still predicted to occur under these interactions. Given our findings, with no discernible difference in temperatures where the onset of bleaching occurs, we expect that coral resistance to thermally induced bleaching is unlikely enhanced by the implementation of protected status. These findings are in accordance with previous studies investigating loss of coral cover within MPAs under climate change [30], and further challenge the assumed benefits of managed resilience for promoting resistance in corals to guide reefs through the gauntlet of climate change [22, 31].

Given the similar bleaching responses between coral reefs residing within and outside MPAs, our findings add to the growing complexity of the relationship between local and global scale stressors for degrading coral reefs. Comparable levels of bleaching where no temperature stress is present (0 DHW category) indicates no difference in bleaching under ambient conditions, which is likely a result of localised conditions [29], survey error [37], and perhaps lack of recovery from a previous disturbance event - i.e. non-branching corals which are able to survive longer while bleached [38]. Furthermore, an identical thermal threshold where the onset of bleaching occurs suggests there is a not a synergistic relationship between local and global factors which exert stress onto coral reefs. Non-synergistic and antagonistic relationships have been widely reported on coral reefs over the last 10 years [32–34] challenging the previous supposition that stressors exerted onto coral reefs act synergistically [39]. Our findings could indicate that bleaching is unlikely to be synergistically exacerbated by local stressors [35], which are assumed to be moderated by MPAs, given the identical bleaching responses between MPA and non-MPA environments (Fig. 4). It is likely that the effects of climate change are far eclipsing the role of localised stressors and thus localised mitigation [11, 22, 40]. It should be noted, however, that many MPAs are not adequately managed in many marine regions [41], and have aims focused on social-economic and biodiversity benefits [42], thus our findings may also reflect this. Furthermore, owing to the spatial variability of coral bleaching [29], which is often specific to a wide range of factors such as turbidity [43, 44], internal waves [45, 46], evolutionary history [47], and ecological memory [48], exceptions to the global scale pattern will exist [20, 21].

Our findings also identify insufficient evidence to support the managed resilience hypothesis for reef corals, because bleaching responses are similar between MPA and non-MPA sites (Figs. 3, 4). Consequently, our findings suggests that the resistance of reef building corals will not be enhanced through the implementation of MPAs, which aim to mitigate local stressors and ameliorate physiological performance of corals [27]. However, it is critical to note there are many other benefits of MPAs which ensure food provision, vital for human livelihood, and maintain biodiversity which is critical for ecosystem function around the globe [23, 24, 42]. Yet, the assumption that MPAs will help support coral reefs by preventing impacts of warming (i.e. bleaching) on corals is likely incorrect based on these findings. Rather, continued warming linked to anthropogenic activity will incessantly bleach corals more often through the Anthropocene [2] regardless of protected status.

## Conclusions

Collectively, our findings add to the growing evidence that protected status will have little impact for alleviating the effects global stressors such as marine heatwaves which will continue to be exerted on coral reefs. While the implementation of effectively designed MPAs can be beneficial for coral cover and maintaining functional species [22, 25], and most critically support communities dependent on coral reefs [49], they will not mitigate the effects of coral bleaching induced by global warming [22, 30]. Consequently, actions targeting the source of rising global temperatures (i.e. greenhouse gas emissions) remains the most effective way to moderate future coral bleaching caused by global warming, and thus mitigating continued global coral reef decline [11, 22, 39, 50].

## Materials and methods

### Bleaching data

Bleaching data were collated from reefcheck.org, combining 8798 surveys from 3,067 sites across 73 countries (Fig. 1), from the years 2002–2018. Validity of the Reef Check data are well established with less than 7% sampling error for identifying components of benthic cover [37]. These data have also been used in previous macroecological coral bleaching studies [29, 44, 51]. From Reef Check data, the percentage of bleached coral populations within each reef check survey (i.e. % reef bleached) were extracted (i.e. site wide bleaching), along with the date of the survey and their geographic coordinates. This allowed for each survey to be spatially and temporally associated with environmental data. The global scale of these data, along with the long term time frame and high sample size provide a robust basis for elucidating relationships between environmental drivers associated with bleaching.

### MPA data

Each Reef Check survey was temporally and spatially associated as being inside or outside an MPA as designated by the IUCNs world data base of protected areas [52] at the time of survey. The reef check survey data were spatially associated by overlaying the MPA shape file with the reef check coordinates using the ‘*sp’* package [53] in R studio 4.0 [54]. From the MPA shape file, all data attributes were extracted for sites which fell within the MPA polygons, summarised in Additional file 1: Table S1. A survey was considered to be in a protected area if it fell within a designated protected region as specified by the IUCN protected area categories [55], shown in Table 2. These protected status categories represent the global definition of a protected area, however all will vary with their level of enforcement, and type of protection incurred. For example, this study has 877 surveys which fell within a protected region that is also a no-take zone, while 1876 surveys within MPAs were not reported (i.e., are unknown) to have a no-take zone. Therefore, for simplicity, we are using the simple definition of whether a survey fell within a protected area to determine how any level of protected status interacts with global warming for influencing coral bleaching.

**Table 2:** IUCN Protected Area Management Category as defined in the IUCN Manual of World Database on Protected Areas User Manual [55]

Code	Protected status category	Number of surveys
Ia	Strict Nature Reserve	67
Ib	Wilderness Area	0
II	National Park	1342
III	National Monument	6
IV	Habitat/Species Management	399
V	Protected Landscape/Seascape	423
VI	Managed Resource Protected Area	368
N/A	Not applicable	675
N/R	Not reported	111

### Environmental data

To determine whether bleaching differs between MPAs and non-MPAs under thermal stress, we used the degree heating weeks (DHWs) metric to quantify thermal anomalies. DHWs represent the global standard for predicting bleaching from thermal stress and are highly robust in their predictions [56]. These data were extracted on a weekly time series from the Coral Reef Temperature Anomaly database (CoRTAD version 6), supplied from the National Oceanic and Atmospheric Administration [57] at a resolution of ~ 4.6 km at the equator. The DHW values were temporally and spatially associated with each coral bleaching survey on a weekly time series using the ‘*ncdf4*’ [58] package in R. DHW are the global standard for determining bleaching likelihood under thermal stress, where one DHW represents 1 °C increase in the local mean climatic temperature for one week over the last 12 weeks.

We included turbidity within our model to account for other environmental drivers which influence coral physiology, and thus bleaching responses [15, 44]. The diffuse attenuation coefficient of light at the 490 nm wavelength is positively related to turbidity, and has been ubiquitously utilised for deriving turbidity measurements in coral reef studies [44, 59, 60]. The kd490 values were extracted from the Modis-Aqua satellite database (https://oceandata.sci.gsfc.nasa.gov/MODIS-Aqua/Mapped/Monthly/4km/Kd_490/) maintained by NASA’s Earth Observation System Data and Information System (EOSDIS). These data were spatially and temporally matched up with each coral bleaching survey on a weekly time series, at a 4 km resolution.

### Statistical analyses

We developed a collinearity matrix using a Pearson’s correlation coefficient on the log transformed MPA attributes (Additional file 1: Fig. S1). We excluded highly collinear data using a conservative 0.65 cut off to prevent collinearity inhibiting convergence of the Bayesian model. If MPA attributes for a survey were missing, the survey was also excluded from the analysis. In total, 3391 Reef Check surveys fell within MPAs at the time of survey which were used in the Bayesian model.

We used a Generalised Linear Model with group specific terms in ‘*rstanarm’* [61], which uses the STAN language [62] in R 4.0 [54]. The response variable, average bleaching prevalence, was modelled with a Negative Binomial distribution as data were dominated by zeros, and is a distribution which has been successfully used on these bleaching data in the past [29]. Coral ecoregions were run as a random effect to account for spatial variation in bleaching patterns (Additional file 1: Fig S4). Coral ecoregions were extracted from Veron et al. [63], Corals Ecoregions of The World (COTW). Any survey which fell outside the ecoregion polygons was excluded from analysis. These coral ecoregions represent consistent patterns of taxonomic configuration, dispersal and isolation processes, and patterns of evolutionary history [63, 64], utilised in multiple global scale coral reef studies to account for spatial variation [29, 44, 59]. The default weakly informative priors which internally adjust in scale based on regression coefficients were used in the model [61]. Predictors were standardised to aid convergence. Posterior predictive checks from the *‘Bayesplot’* package [65] in R were used to assess for model fit (Additional file 1: Fig S2). Convergence was visually assessed through trace plots (Additional file 1: Fig. S3) and achieved when the rhat value (Gelman-Rubin statistic) reached 1.0 [61]. The model ran with 4 chains, for 5,000 iterations with 2500 warmups.

Differences in bleaching prevalence between MPA and non-MPA sites were statistically analysed by transformation of bleaching prevalence data into negative binomial distributions using the ‘*fitdist*’ function in the ‘MASS’ package [36]. The average bleaching prevalence data were split into 4 groupings of thermal stress thresholds of none (0), low (0–1.49), medium (1.5–3.9), and high (> 4) DHW. Bleaching prevalence distributions for each DHW category were then analysed with a likelihood ratio test with 2 degrees of freedom and a true lower tail [29, 44]. Finally, differences in the DHW values associated with bleaching at MPA and non-MPA sites were also statistically analysed with a Likelihood ratio test. The DHW values were fit into a gamma distribution from the ‘MASS’ package. The DHW values > 12 were removed from analysis here, with a constant of 1 also added so data could fit the gamma distribution [36]. The degrees of freedom of the likelihood ratio test were again 2, with the lower tail specified as true.

## Supplementary Information

**Additional file 1. Table S1.** Environmental covariates and MPAattributes used in this study, with a definition and reference to their source. **Fig. S1** Collinearity matrixof explanatory variables for coral bleaching from the Coral Reef TemperatureAnomaly Database (CoRTAD V6) and the World Database of Protected Areas (WDPA).Attributes with 0.65 collinearity score or higher were excluded from the Bayesianmodel. **Fig. S2** Posterior predictivechecks of Bayesian model fit with our bleaching data. **Figure S3**. Trace plots after burnins discardedfrom the Bayesian Generalized linear model with group specific terms ranthrough STAN for each covariate analyzed in the model. **Figure S4**. Intercept variance for each ecoregionran as a random effect within the STAN Bayesian model.

## Data Availability

All data and code used in this study are available on our Github repository (https://github.com/JackVJohnson/Bleaching-in-MPAs). All data used in this study are also publically available from cited sources in the methods.
