# Correction: Marine protected areas do not buffer corals from bleaching under global warming

**DOI:** 10.1186/s12862-022-02034-5

**Published:** 2022-06-21

**Authors:** Jack V. Johnson, Jaimie T. A. Dick, Daniel Pincheira-Donoso

**Affiliations:** 1 Macrobiodiversity Lab, School of Biological Sciences, Queen’s University Belfast, 19 Chlorine Gardens, Belfast, BT9 5DL UK; 2 Institute for Global Food Security, School of Biological Sciences, Queen’s University Belfast, 19 Chlorine Gardens, Belfast, BT9 5DL UK

## Correction: BMC Ecology and Evolution (2022) 22:58 https://doi.org/10.1186/s12862-022-02011-y

Following the publication of the original article [1], we were notified that the authors had overlooked (and thus, failed to explicitly acknowledge) the source for the code availability which facilitated the production of Fig. 1 and data collection in the study. This is:

"The code employed to produce figure 1, and to access CoRTAD and MODIS data used in this study is available from Robert van Woesik and Shannon Sully (https://github.com/InstituteForGlobalEcology).”

The original article has been corrected.
