# Supplementary material for: Marine protected areas do not buffer corals from bleaching under global warming
Source: BMC Ecol Evol. 2022 May 4;22:58. doi: 10.1186/s12862-022-02011-y (PMC9066861; doi:10.1186/s12862-022-02011-y)
Supplement: Supplementary file 1 — Additional file 1. Table S1. Environmental covariates and MPAattributes used in this study, with a definition and reference to their source. Fig. S1 Collinearity matrixof explanatory variables for coral bleaching from the Coral Reef TemperatureAnomaly Database (CoRTAD V6) and the World Database of Protected Areas (WDPA).Attributes with 0.65 collinearity score or higher were excluded from the Bayesianmodel. Fig. S2 Posterior predictivechecks of Bayesian model fit with our bleaching data. Figure S3. Trace plots after burnins discardedfrom the Bayesian Generalized linear model with group specific terms ranthrough STAN for each covariate analyzed in the model. Figure S4. Intercept variance for each ecoregionran as a random effect within the STAN Bayesian model. [file 12862_2022_2011_MOESM1_ESM.docx]

**Supplementary material**

Table S1. Environmental covariates and MPA attributes used in this study, with a definition and reference to their source.

| **Attribute** | **Definition** | **Source** |
| --- | --- | --- |
| SSTA_DHW | SSTA_DHW, sum of previous 12 weeks when SSTA is greater than or equal to 1 degree C. | <https://www.ncei.noaa.gov/products/coral-reef-temperature-anomaly-database> |
| SSTFilled | The weekly average SST value in Kelvin |  |
| MPA_AGE | The calculated age based on the year the protected region was implemented, minus the year of the reef check survey. | <https://www.iucn.org/theme/protected-areas/our-work/world-database-protected-areas> |
| REP_M_AREA | Covers the extent of the protected area which falls within the marine environment in square kilometres. Information is supplied by the data provider for the specified protected area. |  |
| GIS_M_AREA | Covers the extent of the protected area which falls within the marine environment in square kilometres. Calculated by UNEP-WCMC using Mollweide projection. |  |
| REP_AREA | Covers the entirety of the protected region (marine, freshwater and terrestrial environments). Supplied by the data provider. |  |
| GIS_AREA | Covers the entirety of the protected region (marine, freshwater and terrestrial environments). Calculated by UNEP-WCMC using Mollweide projection. |  |
| NO_TK_AREA | The area within the marine environment designated as a no-take zone. Reported by the data provider. |  |
| Kd490_value | The light attenuation coefficient as a measure of turbidity. | <https://oceandata.sci.gsfc.nasa.gov/MODIS-Aqua/Mapped/Monthly/4km/Kd_490/> |


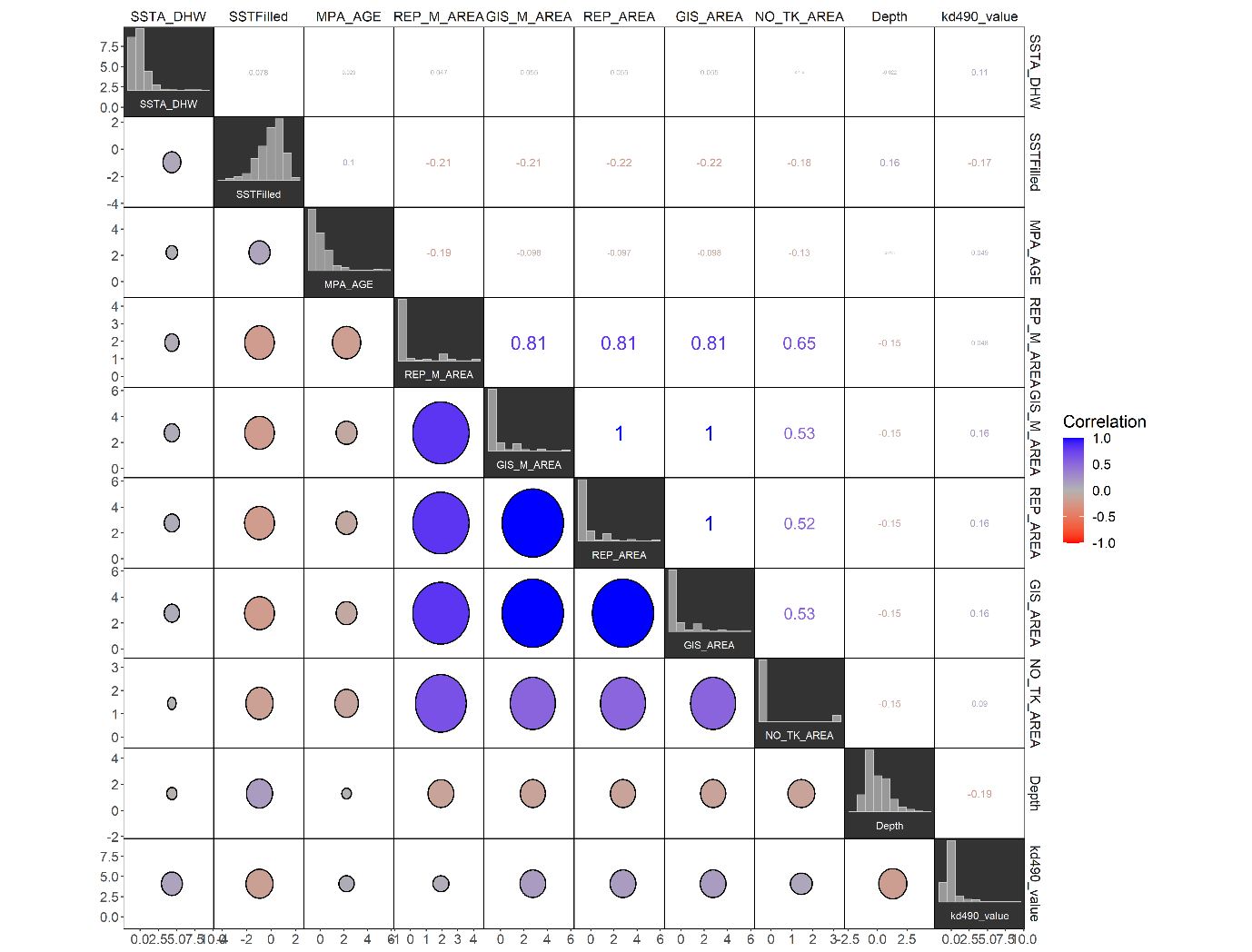


**Fig. S1** Collinearity matrix of explanatory variables for coral bleaching from the Coral Reef Temperature Anomaly Database (CoRTAD V6) and the World Database of Protected Areas (WDPA). Attributes with 0.65 collinearity score or higher were excluded from the Bayesian model.


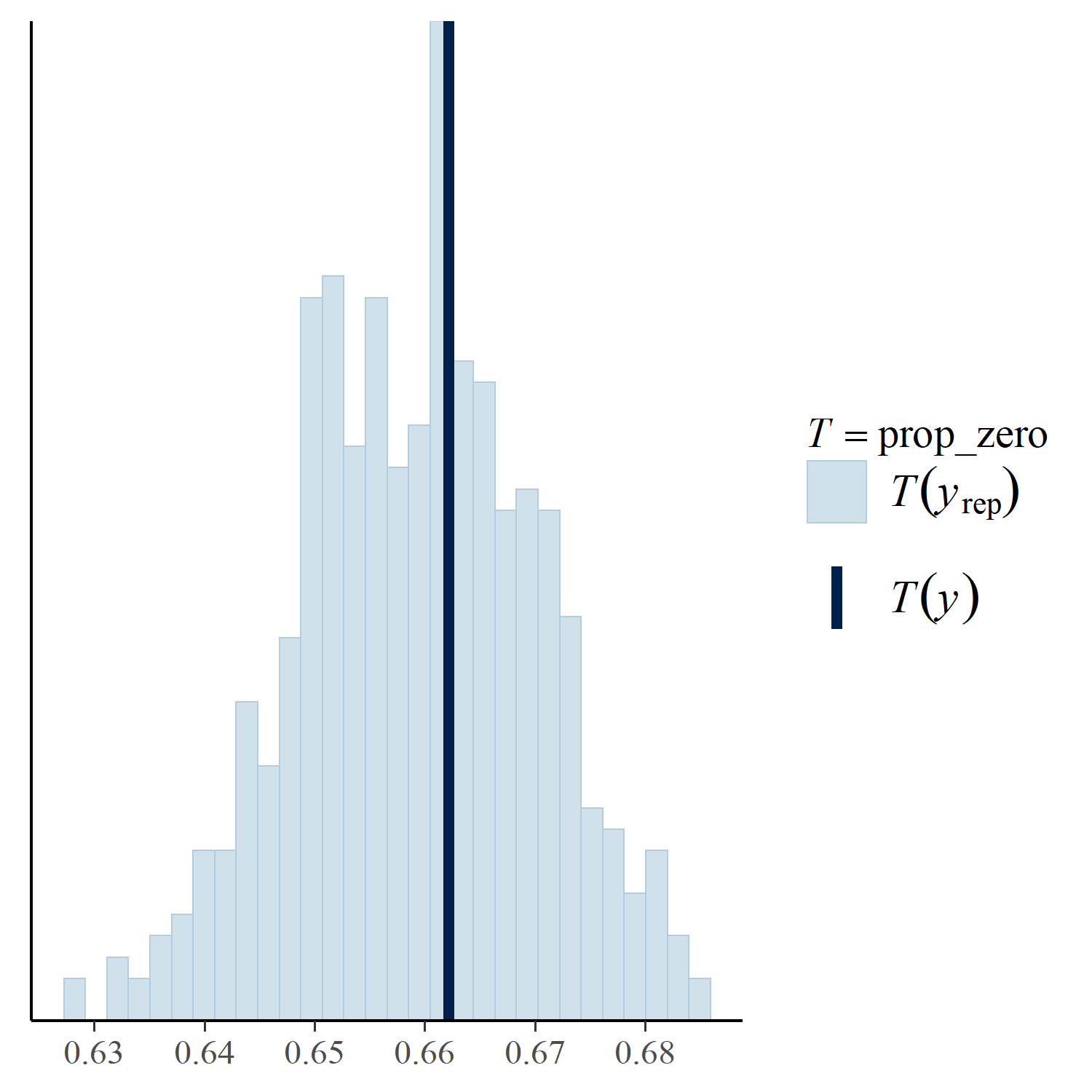


**Fig. S2** Posterior predictive checks of Bayesian model fit with our bleaching data.


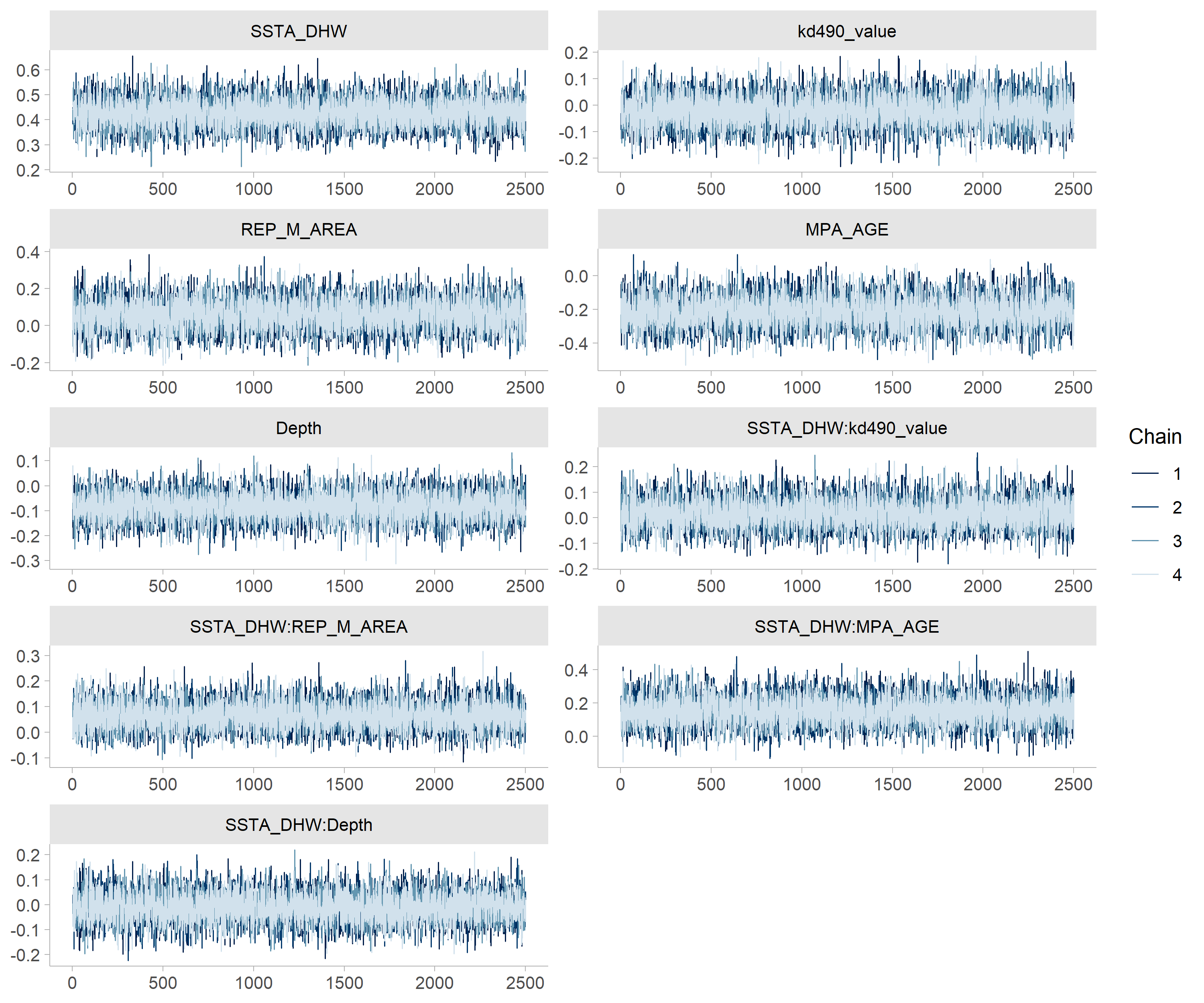


Figure S3. Trace plots after burnins discarded from the Bayesian Generalized linear model with group specific terms ran through STAN for each covariate analyzed in the model.


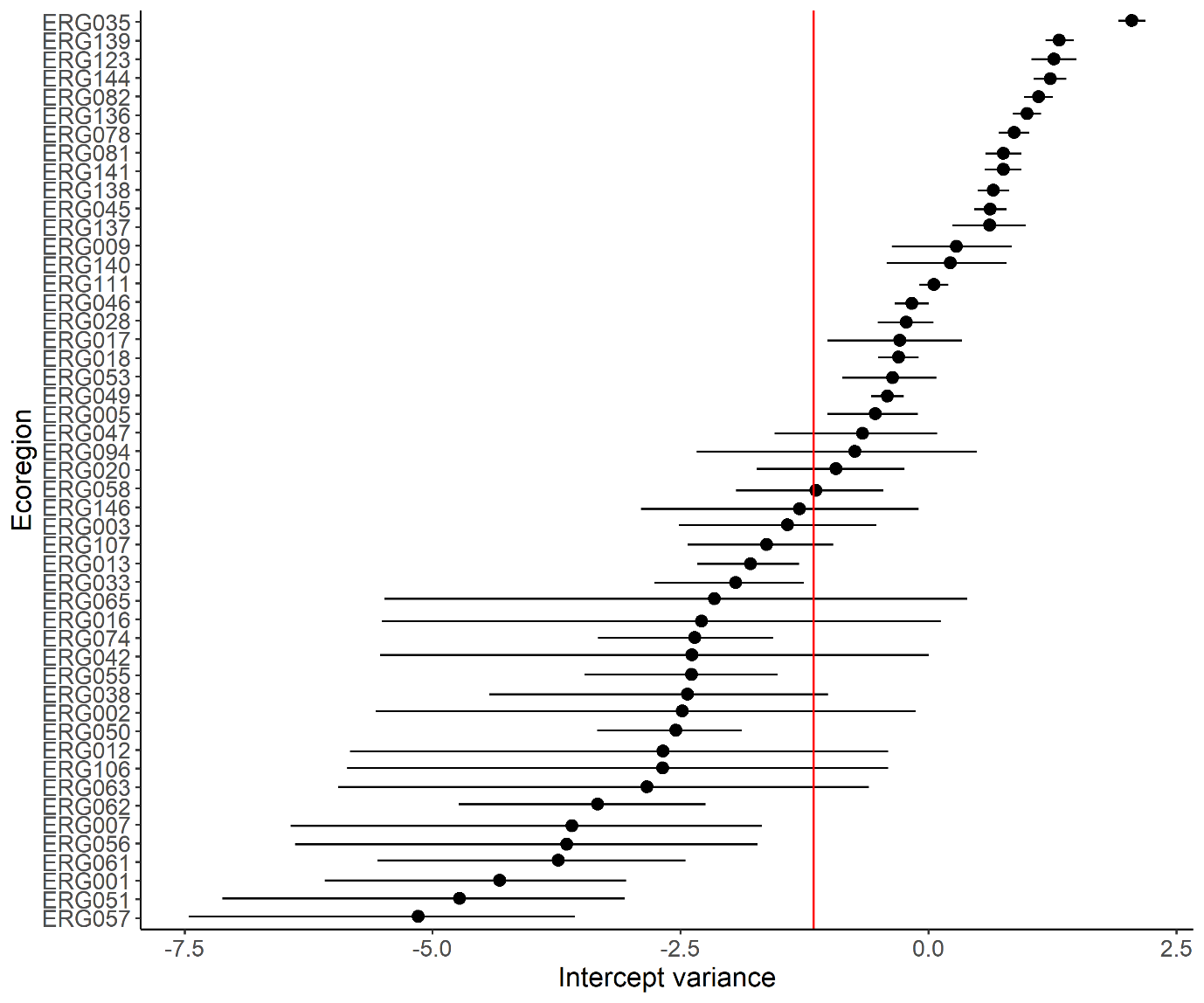


Figure S4. Intercept variance for each ecoregion ran as a random effect within the STAN Bayesian model.
